# Relations among perceived stress, fatigue, and sleepiness, and their effects on the ambulatory arterial stiffness index in medical staff: A cross-sectional study

**DOI:** 10.3389/fpsyg.2022.1010647

**Published:** 2022-10-27

**Authors:** Xiaorong Lang, Quan Wang, Sufang Huang, Danni Feng, Fengfei Ding, Wei Wang

**Affiliations:** ^1^School of Nursing, Tongji Medical College of Huazhong University of Science and Technology, Wuhan, China; ^2^Tongji Hospital Affiliated with Tongji Medical College of Huazhong University of Science and Technology, Wuhan, China; ^3^Department of Pharmacology, Fudan University Basic Medicine College, Shanghai, China

**Keywords:** perceived stress, fatigue, sleepiness, ambulatory arterial stiffness index, medical staff, path analysis

## Abstract

**Objective:**

To explore the relations among perceived stress, fatigue, sleepiness, and the pathway of their effects on the ambulatory arterial stiffness index (AASI) among medical staff.

**Methods:**

This cross-sectional study was conducted at a tertiary hospital in Wuhan, China. Perceived stress, fatigue, and sleepiness were measured using the perceived stress scale (PSS), Fatigue assessment scale (FAS), and Epworth Sleepiness Scale (ESS), respectively. AASI was obtained from 24-h ambulatory blood pressure monitoring. Path analysis was used to clarify the relations among the PSS, FAS, and ESS scores, and their relations to AASI values.

**Results:**

A total of 153 participants were included herein. The PSS and FAS correlated with the ESS (*r* = 0.424, *p* < 0.001), and the PSS correlated with the FAS (*r* = 0.614, *p* < 0.001). In addition, the ESS correlated with the AASI (*r* = 0.225, *p* = 0.005). According to the path analysis results, the PSS and FAS had no direct effect on the AASI, but did have an indirect effect on this index (β = 0.059, 95% confidence interval [CI] = 0.017–0.128, *p* = 0.005; β = 0.059, 95%CI = 0.016–0.135, *p* = 0.006, respectively) by influencing the ESS (β = 0.263, β = 0.262, *p* = 0.004).

**Conclusion:**

Sleepiness was a mediator of the effects of perceived stress and fatigue on AASI.

## Introduction

As the main providers of medical services, medical staff have primary responsibility for patient recovery. The coronavirus disease of 2019 (COVID-19), which initially broke out in December 2019, has negatively impacted many countries, dramatically changed people’s lives, and increased work challenges for medical staff (Haleem et al., 2020). Social and work environment risk factors can have profound adverse effects on mental health among hospital staff (Greenberg et al., 2020; Kapetanos et al., 2021; Saeed et al., 2021). One epidemiological survey showed that medical workers in China displayed significant psychological disturbance including anxiety symptoms, depression symptoms, stress-related symptoms, and sleep problems, with aggregate prevalence rates of 27.0, 26.2, 42.1, and 34.5%, respectively, during the COVID-19 outbreak (Zhang et al., 2021). Moreover, the COVID-19 pandemic can now also be considered a chronic stressor for medical staff, with multiple negative effects that include an unsustainable workload, excessive financial hardship, and fear of uncertainty regarding continued impact (Gupta et al., 2021). Therefore, psychological disorders among medical staff may be persistent, and attention should be paid to their mental health.

Perceived stress, defined as “the degree to which situations in one’s life are appraised as stressful,” refers to feelings of unpredictability, uncontrollability, and overload (Lehrer et al., 2020). One study found a prevalence of high perceived stress as high as 56% among medical staff in the COVID-19 context (Yan et al., 2021). Physiologically, high stress levels activate the autonomic nervous system and hypothalamic–pituitary–adrenal axis, reduce immunity, and increase inflammatory cytokines, which can lead to physiological changes, including cardiovascular system and sleep behavior alterations (Kemeny, 2003). Furthermore, higher perceived stress is associated with sleepiness and can lead to risk for adverse cardiovascular outcomes (Cummings et al., 2016; Valente et al., 2019).

Sleepiness, a consequence of disordered, poor, and insufficient sleep, is defined in the MeSH database as a compelling urge to sleep. The main causes of sleepiness are social environment (e.g., the COVID-19 pandemic), psychological distress, poor mental health, insufficient sleep, and disease (e.g., obstructive sleep apnea) (Becerra et al., 2022; Thorarinsdottir et al., 2022). A link between daytime sleepiness and cardiovascular diseases, including hypertension and stroke—which are related to arterial stiffness—is well established (Yang et al., 2022).

The ambulatory arterial stiffness index (AASI), defined as 1 minus the regression slope of blood pressure (BP) values obtained by 24-h ambulatory BP monitoring (ABPM), is a relatively new indicator of arterial stiffness (Li et al., 2006). In addition to reflecting degree of atherosclerosis, AASI can predict subclinical left ventricular (LV) systolic dysfunction and is an independent predictor of major adverse cardiovascular events (Ahmed et al., 2020; Boos et al., 2021). It is thus considered an important and promising risk prediction tool.

Fatigue, defined as “a subjectively unpleasant symptom that incorporates total body feelings ranging from tiredness to exhaustion, creating an unrelenting overall condition which interferes with individuals’ ability to function at normal capacity” (Ream and Richardson, 1996), is common among medical staff due to their work environment and pressures. Chronic fatigue and adverse cardiovascular events are associated (Naess et al., 2005) and fatigue is linked to sleepiness (Kim et al., 2019), which indicates poor health (Becerra et al., 2022). Thus, fatigue may also lead to a decline in work quality among medical staff. Moreover, the positive association between higher perceived stress and greater fatigue symptoms is also well-established (Wang et al., 2021). Cumulatively, fatigue may moderate the relation between perceived stress and sleepiness; it may also moderate the indirect effects of perceived stress on AASI, through sleepiness.

Although numerous studies have examined perceived stress, fatigue, and drowsiness among health professionals, to our knowledge none have linked these factors to AASI. Therefore, our aim was to clarify the associations among perceived stress, fatigue, and drowsiness, and their relations to AASI. We proposed the following hypotheses: (1) perceived stress, fatigue, and sleepiness are intercorrelated; (2) perceived stress and fatigue affect sleepiness; (3) sleepiness affects AASI; (4) perceived stress and fatigue directly and/or indirectly affect AASI; and (5) sleepiness mediates the effects of perceived stress and fatigue on AASI.

## Materials and methods

### Study design

This cross-sectional study was conducted at a large general hospital in Wuhan, China. This study was approved by the Ethics Committee of the Tongji Medical College of Huazhong University of Science and Technology, with IRB approval number 2021S141.

### Participants and procedures

Participants, selected by convenience sampling, were asked to fill out questionnaires and scales through the Research Electric Data Capture platform. They were invited to participate if they: (1) did not have any plans to leave their current position within 6 months; (2) had no serious medical condition (e.g., cancer, stroke); and (3) were willing to participate and sign the informed consent form. All participants were informed of the study aims and methods to maintain authenticity and anonymity before they signed informed consent.

### Sample size calculation

We used an online site^1^ based on Andrew Fisher’s formula to estimate the required sample size for sufficient power. According to a previous study, the average standard deviation of AASI values ranges from 0.06 to 0.22 in the general population (i.e., those with or without cardiovascular diseases) (Kollias et al., 2012). Based on this, we calculated the needed sample size as between 86 and 264 using the formula (sample size=$\frac{Z_{\alpha }^{2}*SD*\left(1-SD\right)}{d^{2}}$) and a 95% confidence level [CI].

### Questionnaires

A structured, standard questionnaire was used to collect medical staff demographics, including age, gender, educational level, marital status, job station, working years, and number of hours worked per week. Body mass index (BMI) was calculated based on participants’ self-reported weight and height, and clinically diagnosed diseases and subjective health status were assessed by specific questions.

### Perceived stress scale

The perceived stress scale (PSS) is a widely used 14-item instrument that assesses stress levels in young people and adults aged 12 and above. Items were designed to tap how unpredictable, uncontrollable, and overloaded respondents find their lives. The questions in the PSS ask about feelings and thoughts during the last month. The overall score ranges from 0 to 56. A score of ≥28 indicates at least moderate stress and higher scores indicate greater perceived stress (Rebello et al., 2018). The Cronbach’s α for this scale is 0.830 among a Chinese population (Huang et al., 2020). Herein, the Cronbach’s α was 0.835.

### Fatigue assessment scale

The Fatigue assessment scale (FAS) is a 10-item self-report scale evaluating symptoms of chronic fatigue. The FAS treats fatigue as a unidimensional construct and does not separate its measurement into different factors. However, to ensure that the scale evaluates all aspects of fatigue, it measures both physical and mental symptoms. The total score ranges from 10 to 50 and higher scores indicate worse fatigue. Scores between 22 and 35 are classified as moderate fatigue, and those >35 indicate substantial fatigue (De Vries et al., 2004; Hendriks et al., 2018). The Cronbach’s α for the Chinese version of the FAS is 0.71–0.82 (Ho et al., 2021). Herein, the Cronbach’s α was 0.883, indicating highly satisfactory internal reliability.

### Epworth sleepiness scale

The Epworth sleepiness scale (ESS), developed at Epworth Hospital in Melbourne, Australia, is an 8-item self-administered questionnaire. Its psychometric properties have been widely investigated. With a score range of 0–24, higher ESS scores indicate greater daytime sleepiness, or a higher sleep propensity, in daily life. A score of >6 points indicates daytime sleepiness and >10 points indicates excessive daytime sleepiness (Johns and Hocking, 1997). The Cronbach’s α for the Mandarin version of the ESS is 0.80 (Wu et al., 2012). Herein, the Cronbach’s α was 0.768, confirming its reliability.

### Ambulatory arterial stiffness index

The AASI is defined as 1 minus the regression slope of diastolic on systolic BP values obtained from 24-h ABPM. ABPM was performed using a noninvasive ABPM instrument manufactured by a durable medical supply company in Beijing, China. BP readings were obtained at 30-min intervals during the daytime (6:30–22:00) and at 60-min intervals during the nighttime (22:00–6:30 the next day). Of the total readings, ≥ 80% were considered valid. AASI was computed using the formula:


$$\mathrm{AASI}=1-\mathrm{slope}\;\left(\mathrm{systolic}\;BP/\mathrm{diastolic}\;BP\right)$$

### Statistical analysis

SPSS version 26.0 was used to calculate descriptive statistics and run correlation analyses. A value of *p* <0.05 (two-tailed) was considered statistically significant. AMOS version 26.0 was used to run path analyses, a subset of structural equation modeling (SEM) used to estimate and assess direct, indirect, and mediation effects among variables, with maximum likelihood estimations for testing our hypothesis. Multiple fit indicators were used to evaluate the model, with the qualified criteria: χ^2^/df < 3, GFI > 0.90, RMSEA < 0.05, RMR < 0.05, CFI > 0.90, NFI > 0.90, and NNFI > 0.90 (Xia and Yang, 2019). The bias-corrected bootstrap confidence interval with 5,000 bootstrap samples was used to evaluate the significance of indirect effects.

## Results

### Participant characteristics

A total of 153 participants were included in the study. Their mean age was 30.46 ± 10.46 years and the sample included 88 (57.52%) nurses, 37 (24.18%) physicians, and 28 (18.30%) other health workers. Table 1 shows detailed characteristics.

**Table 1:** Participant characteristics.

Characteristic	Category	*N* (mean)	% (SD)
Age		30.46	10.46
Gender	Male	38	24.84
	Female	115	75.16
Educational level	Undergraduate or below	15	9.80
	Postgraduate or above	138	90.20
Marital status	Unmarried	43	28.10
	Married	110	71.90
Station	Nurse	88	57.52
	Physician	37	24.18
	Other healthcare workers	28	18.30
Years working		9.67	6.38
Weekly working hours		46.55	11.49
Working night shift	No	15	9.80
	Yes	138	90.20
BMI		19.87	6.81
Diagnosed illness	No	128	83.66
	Yes	25	16.34
Self-perceived health level	Poor	33	21.57
	So-so	101	66.01
	Good	19	12.42

### Descriptive data, correlations, and multi-collinearity

According to Table 2, average scores were 31.16 ± 6.83 for the PSS, 30.42 ± 5.95 for the FAS, and 8.84 ± 3.99 for the ESS. The AASI values were 0.38 ± 0.19. As presented in Table 3, both the PSS and FAS were correlated with the ESS (*r* = 0.424, *p* < 0.001) and the PSS was correlated with the FAS (*r* = 0.614, *p* < 0.001). In addition, the ESS was correlated with the AASI (*r* = 0.225, *p* = 0.005). Though intercorrelations were strong, VIF values were < 5 and tolerance values were > 0.2, indicating no multi-collinearity between variables.

**Table 2:** PSS, FAS, ESS, and AASI values.

Variable	Mean	SD
PSS	31.16	6.83
FAS	30.42	5.95
ESS	8.84	3.99
AASI	0.38	0.19

**Table 3:** Correlations and multi-collinearity values for PSS, FAS, ESS, and AASI.

Variables	PSS	*p*-Value	FAS	*p*-Value	ESS	*p*-Value	VIF	Tolerance
PSS	1						1.695	0.590
FAS	0.614	<0.001	1				1.695	0.590
ESS	0.424	<0.001	0.424	<0.001	1		1.287	0.777
AASI	0.129	0.111	0.024	0.765	0.225	<0.001	–	–

### Path analysis

**Figure 1:**
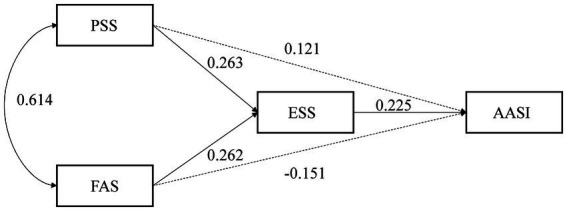
Model diagram of ESS as a mediator variable.

We first considered a model with the ESS as a partial mediator, that is, that the PSS and FAS affect the AASI both directly and through the ESS. However, the analysis showed that neither the PSS (β = 0.121, *p* = 0.235) nor the FAS (β = −0.151, *p* = 0.139) had a significant effect on the ESS, which was represented by dashed lines in Figure 1, indicating a lack of support for the partial mediation model. Thus, the analysis was repeated with the ESS as a full mediator. The level of fit for this mediating model was satisfactory: χ^2^/df = 1.199; GFI = 0.992; RMSEA = 0.036; RMR = 0.028; CFI = 0.997; NFI = 0.980; and NNFI = 0.927. The final model diagram and effects estimate are shown in solid lines in Figure 1 and Table 4. The PSS and FAS directly affected the ESS (β = 0.263, β = 0.262, respectively, both *p* = 0.004). The ESS directly affected the AASI (β = 0.225, *p* = 0.004). The PSS and FAS had indirect effects on the AASI (β = 0.059, 95%CI = 0.017–0.128, *p* = 0.005; β = 0.059, 95%CI = 0.016–0.135, *p* = 0.006, respectively), and the ESS mediated the PSS and FAS.

**Table 4:** Effects estimate of hypothesized model (standardized coefficients).

Structural path	Direct effect	*p*-Value	Indirect effect (95%CI)	*p*-Value
PSS → ESS	0.263	0.004	–	–
FAS → ESS	0.262	0.004	–	–
ESS → AASI	0.225	0.004	–	–
PSS → AASI	–	–	0.059 (0.017–0.128)	0.005
FAS → AASI	–	–	0.059 (0.016–0.135)	0.006

## Discussion

Herein, we found that perceived stress, fatigue, and sleepiness are common among medical staff. About 58.17% of our sample had at least moderate stress, and 36.64% had excessive daytime sleepiness, similar to previous reports (Busch et al., 2021; Kowalczuk et al., 2021). Furthermore, 92.16% reported at least moderate fatigue, which may be due to the fact that 75.16% of our study was female. Certain sex-specific physiologic factors (e.g., menstruation, contraception) and social background combined with living conditions (e.g., balancing employment and childcare) can cause women to report fatigue more often compared with men (Bensing et al., 1999). Hence it is important to focus on mental health among medical staff, especially in the COVID-19 context.

Perceived stress, fatigue, and sleepiness were significantly intercorrelated, and perceived stress and fatigue were both positively related to sleepiness, supporting our first two hypotheses. These findings are also theoretically sound. According to Hans Selye, stress is the non-specific response to environmental stimuli, including general adaptation syndrome and local adaptation syndrome (Selye, 1976). General adaptation disorders include several common symptoms and signs, including fatigue, sleep problems, and gastrointestinal syndrome (Selye, 1998). The effect of fatigue on sleepiness might be *via* inhibition of the central nervous system. An accumulation of brain adenosine causes tiredness, which, with further sleep deprivation, can manifest as mental exhaustion. This feedback loop can lead to the central nervous system activating protective mechanisms to avoid further damage from excessive fatigue, activating movement and nerve inhibition and a decline in physical vitality, or a tired response (Martin et al., 2018). Fatigue has an inhibitory effect on neurons, leading to difficulty with maintaining active cognitive regulation; consequently, neuroendocrine interactions help systematically reallocate cognitive resources in response to stress (Hermans et al., 2014). This leads to the long-term experience of negative emotions; the significant burden of stress on cognitive emotion regulation may thus increase stress-related emotional sensitivity and intensity (Weymar et al., 2012).

Moreover, the mean ESS score among our participants was 8.84 points; that it was >6 indicates common daytime sleepiness among these medical staff. Daytime sleepiness is associated with development of cardiometabolic disease (Qureshi et al., 1997; Newman et al., 2000; Jia et al., 2022), consistent with our result and supporting our third hypothesis. In terms of mechanism of action, sleep disorders diminish nitric oxide bioavailability to impair nitric oxide-mediated endothelial-dependent vasodilation, leading to a hardening of the arteries and increased risk of cardiovascular disease (Bain et al., 2017).

However, we did not find direct effects of either perceived stress or fatigue on AASI. Previous studies have also found inconsistent relations between perceived stress and cardiovascular reactivity (Klatzkin et al., 2019). For example, Steptoe and Kivimäki reviewed the literature to find an inconclusive relation between stress and stroke; in particular, self-reported stress was not a strong predictor of stroke (Steptoe and Kivimäki, 2013). Yet it is worth noting that there is a gender difference in the association between fatigue and cardiovascular disease, with a stronger causal relation in males than in females (Honkonen et al., 2006), which might explain why fatigue did not directly affect AASI in our study.

Though the average PSS score herein was 31.16, representing moderate stress, we believe the lack of association with AASI may be due to the fact that our participants are relatively young and thus have some regulatory flexibility to reduce the cumulative burden of stress, so that it did not translate directly to cardiovascular risk. Another possibility is that, consistent with Dienstbier’s model of physiological resilience, exposure to moderate life stress may cause individuals to adapt or ‘toughen’ and show an adaptive physiological response to acute challenges, and then recover relatively quickly (Dienstbier, 1989). Although the average FAS score herein was 30.42, indicating moderate fatigue among these medical staff, most participants were women. The effects of fatigue on the cardiovascular system is due to a chronic, long-term process (i.e., unexplained fatigue lasting at least 6 months) (Natelson et al., 2021), possibly explaining why fatigue in our sample did not have a significant direct effect on AAS. Thus, the current perceived stress and fatigue among this sample may not pose a health threat.

Nevertheless, the effect of a stress–fatigue interaction on health should not be ignored. According to our SEM analysis, fatigue played a moderating role between perceived stress and sleepiness, and also influenced AASI indirectly *via* sleepiness symptoms. If we consider AASI as a predictor of LV systolic dysfunction, medical staff who are both stressed and fatigued may be at higher risk for developing cardiovascular disease. Therefore, hospital administrators and managers should consider monitoring stress and fatigue symptoms among their medical staff, and then implementing effective interventions.

There were some study limitations. First, ours was a single-center study at a tertiary hospital, which may limit its generalizability. It will therefore be necessary to carry out multicenter studies, including multi-level hospitals, to draw more accurate conclusions. Second, this was a cross-sectional study, so that causation will need to be established with future prospective studies. Finally, though based on an *a priori* power analysis, our sample size was still relatively small and future studies should include larger samples.

## Conclusion

The findings herein indicate that perceived stress, fatigue, and sleepiness are significantly intercorrelated, and that fatigue has a positive effect on perceived stress. Furthermore, while sleepiness has a significant effect on AASI, neither perceived stress nor fatigue have direct effects on AASI. Cumulatively, sleepiness is an intermediate variable between AASI and both perceived stress and fatigue.

## Data availability statement

The raw data supporting the conclusions of this article will be made available by the authors, without undue reservation.

## Ethics statement

The studies involving human participants were reviewed and approved by the Ethics Committee of Tongji Medical College of Huazhong University of Science and Technology. The patients/participants provided their written informed consent to participate in this study.

## Author contributions

XL performed the data analysis, drafted the initial manuscript, and made subsequent revisions. QW designed the study and revised the manuscript. SH reviewed and provided feedback on the manuscript. DF was involved in data collection. FD and WW led the development and advancement of the research project. All authors contributed to the article and approved the submitted version.

## Funding

This study received funding from the National Key R&D Program of China (Program ID: 2021YFC2502200) and National Natural Science Foundation of China (no. 71874063), and partially supported by the collaborative research project between Tongji Hospital and HUAWEI Technologies Terminal Co., Ltd., Shenzhen, China (YBN2019085075).

## Conflict of interest

The authors declare that this study received funding from HUAWEI Technologies Terminal Co., Ltd. The funder had the following involvement with the study: device support.

Other funders were not involved in the study design, collection, analysis, interpretation of data, the writing of this article or the decision to submit it for publication.

## Publisher’s note

All claims expressed in this article are solely those of the authors and do not necessarily represent those of their affiliated organizations, or those of the publisher, the editors and the reviewers. Any product that may be evaluated in this article, or claim that may be made by its manufacturer, is not guaranteed or endorsed by the publisher.

## Footnotes

^1^ https://www.geopoll.com/blog/sample-size-research/
