# Corrigendum: Relations among perceived stress, fatigue, and sleepiness, and their effects on the ambulatory arterial stiffness index in medical staff: A cross-sectional study

**DOI:** 10.3389/fpsyg.2022.1117115

**Published:** 2023-01-04

**Authors:** Xiaorong Lang, Quan Wang, Sufang Huang, Danni Feng, Fengfei Ding, Wei Wang

**Affiliations:** ^1^School of Nursing, Tongji Medical College of Huazhong University of Science and Technology, Wuhan, China; ^2^Tongji Hospital Affiliated with Tongji Medical College of Huazhong University of Science and Technology, Wuhan, China; ^3^Department of Pharmacology, Fudan University Basic Medicine College, Shanghai, China

**Keywords:** perceived stress, fatigue, sleepiness, ambulatory arterial stiffness index, medical staff, path analysis

In the published article, there was an error in affiliation 1. Instead of “School of Nursing, Tongji Medical College of Huazhong University of Science and Technology; Tongji Hospital Affiliated with Tongji Medical College of Huazhong University of Science and Technology, Wuhan, China” it should be “School of Nursing, Tongji Medical College of Huazhong University of Science and Technology, Wuhan, China.”

There was also an error in the text. Due to a clerical error, the number of hospitals in the study was incorrectly stated.

A correction has been made to *Materials and methods, Study design*. The incorrect sentence previously stated: “This cross-sectional study was conducted at two large general hospitals in Wuhan, China.”

The corrected paragraph appears below.

This cross-sectional study was conducted at a large general hospital in Wuhan, China. This study was approved by the Ethics Committee of the Tongji Medical College of Huazhong University of Science and Technology, with IRB approval number 2021S141.

The authors apologize for these errors and state that they do not change the scientific conclusions of the article in any way. The original article has been updated.

